# A Lightweight Cross-Layer Smoke-Aware Network

**DOI:** 10.3390/s24134374

**Published:** 2024-07-05

**Authors:** Jingjing Wang, Xinman Zhang, Cong Zhang

**Affiliations:** 1 School of Automation Science and Engineering, Xi’an Jiaotong University, Xi’an 710049, China; wjingjing@stu.xjtu.edu.cn; 2 AECC Sichuan Gas Turbine Establishment, Mianyang 621000, China; asccwish@163.com

**Keywords:** smoke detection, CLSANet, cross-layer connection, spatial perception, texture federation, self-collaboration head

## Abstract

Smoke is an obvious sign of pre-fire. However, due to its variable morphology, the existing schemes are difficult to extract precise smoke characteristics, which seriously affects the practical applications. Therefore, we propose a lightweight cross-layer smoke-aware network (CLSANet) of only 2.38 M. To enhance the information exchange and ensure accurate feature extraction, three cross-layer connection strategies with bias are applied to the CLSANet. First, a spatial perception module (SPM) is designed to transfer spatial information from the shallow layer to the high layer, so that the valuable texture details can be complemented in the deeper levels. Furthermore, we propose a texture federation module (TFM) in the final encoding phase based on fully connected attention (FCA) and spatial texture attention (STA). Both FCA and STA structures implement cross-layer connections to further repair the missing spatial information of smoke. Finally, a feature self-collaboration head (FSCHead) is devised. The localization and classification tasks are decoupled and explicitly deployed on different layers. As a result, CLSANet effectively removes redundancy and preserves meaningful smoke features in a concise way. It obtains the precision of 94.4% and 73.3% on USTC-RF and XJTU-RS databases, respectively. Extensive experiments are conducted and the results demonstrate that CLSANet has a competitive performance.

## 1. Introduction

Smoke detection, as an early warning of flames, is extremely essential to minimize the damage to ecosystems, public safety, and human life and property caused by fires. Previous smoke detection approaches rely heavily on manual observation or specialized smoke sensors, but they are inevitably constrained by the limited human resources and monitoring range, respectively [1]. In recent years, thanks to the mature installation of surveillance cameras and the Internet of Things (IoT) formed by millions of smart edge devices, vision-based smoke detection, as a study focus, has gradually attracted wide public attention [2].

To implement smoke detection in practical production, many researchers have devoted great efforts and a number of advanced technologies have been developed. Based on the traditional features of smoke, Tian et al. [3] devised an image formation model formulated as convex optimization that solved a sparse representation problem using dual dictionaries for the smoke and background components, respectively. Dimitropoulos et al. [4] presented a new higher-order linear dynamical system (h-LDS) descriptor, which applied higher-order decomposition of the multidimensional image data and dynamic analysis to a video-based smoke identification system. With the development of deep learning, Yar et al. [5] employed a novel vision transformers (ViT) architecture by combining shifted patch tokenisation and local self-attention modules, but it only implements fire scene classification and has no localization capability. Tao et al. [6] gave an adaptive frame selection network (AFSNet) with augmented dilated convolution for smoke detection. The pixels with high responses and the local regions were taken into account to learn the contribution of each pixel automatically. Cao et al. [7] proposed an enhanced feature foreground network (EFFNet). It utilized efficient branch channels to predict the source mask and bounding boxes of smoke plumes. However, smoke detection is still a challenging task due to its irregular shape, complex state, and appearance which are susceptible to environmental disturbances.

Cross-layer connection can enhance the original features of each layer by enabling information exchange among multiple layers and is progressively applied to visual inspection to strengthen performance. Feature pyramid networks (FPNs) and path aggregation networks (PANs) [8], as classical cross-layer structures, fused semantic and object details over features at different scales in an incremental manner. Moreover, Li et al. [9] presented a cross-layer extraction structure and multi-scale down-sampling network with bidirectional transpose FPN (BCMNet) for smoke detection, and its cross-layer incorporated linear feature multiplexing and receptive field amplification. Long et al. [10] added horizontal and vertical connections to a regularized cross-layer ladder network (RCLN), where all four layers of features were computed by manifold regularization constraints to maximize information transmission. Li et al. [11] devised a cross-layer feature pyramid network (CFPN). It indiscriminately aggregated multi-scale features from different levels to gain access to rich contexts. As we can see, the existing cross-layer connectivity regards each feature map equally, i.e., it conducts the same operations on each layer during fusion but ignores the differences between high- and low-level features, which introduces a certain degree of information redundancy and noise in the extracted features. Considering the variability of smoke, it is tough to design an effective cross-layer network to integrate its features.

**Figure 1:**
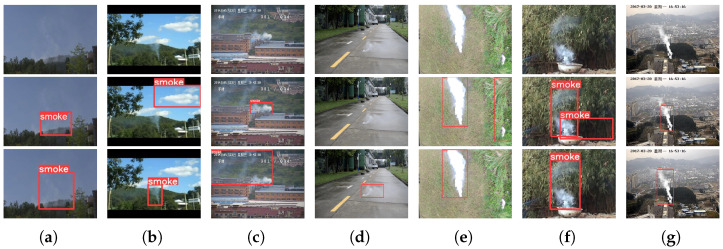
Examples of the input images and their detection results of different algorithms, which are (**a**) SSD [12], (**b**) RetinaNet-50 [13], (**c**) RetinaNet-101 [13], (**d**) EfficientDet [14], (**e**) YOLOv5 [15], (**f**) YOLOX [16], and (**g**) EdgeYOLO-Tiny [17], respectively. Each column includes the input image and its associated detection results obtained by the existing approach and our CLSANet. It is apparent that the conventional methods inevitably suffer from false and missed detection, but our proposed CLSANet can distinguish interference and detect precise smoke areas, which demonstrates the effectiveness of our cross-layer design.

In this paper, we propose a lightweight cross-layer smoke-aware network (CLSANet) with only 2.38 M, and it shares information across different levels with varying preferences to enhance feature propagation and improve real-world smoke detection. To the best of our knowledge, a significant difference between CLSANet and existing approaches is that CLSANet does not have an equal but a different focus on the treatment of high- and low-level features when deploying cross-layer connectivity. Specifically, we only embed the spatial details derived from the low layer and the semantic features developed from the high layer in each fusion stage to preserve the favorable information. Following this principle, the design of our CLSANet performs biased cross-layer cooperation of features between the high and low levels, which effectively removes redundancy and retains meaningful details. We compare the smoke detection results of different algorithms, including SSD [12], RetinaNet-50 [13], RetinaNet-101 [13], EfficientDet [14], YOLOv5 [15], YOLOX [16], EdgeYOLO-Tiny [17], and CLSANet, as shown in Figure 1. To facilitate comparison, the original images are presented in the first row, the second row gives the detection results of the existing algorithms, and the third row provides the performance of our CLSANet. When confronted with smoke in various forms, such as light smoke, fuzzy smoke, or interference from smoke analogs, existing methods suffer from inaccuracies such as missed or false detection. It can be found that the proposed CLSANet shows the most sensitive and accurate detection of smoke, indicating the superiority of our biased cross-layer design. The main contributions of this paper are as follows:

1. A lightweight cross-layer smoke-aware network (CLSANet) which is only 2.38 M is designed. Considering the variable shape and appearance of smoke, it integrates and distributes cross-scale features with distinct preferences and encourages dynamic communication across layers.
2. We propose a spatial perception module (SPM) to enable low-level spatial information to guide the refinement of semantic features in deep layers. The separate features of each layer can thus access both spatial and semantic details, resulting in a decreased loss of important information during the multi-layer progressive fusion.
3. We propose a texture federation module (TFM) as the final feature encoding. Spatial texture attention (STA) is designed to filter shallow information, and at the high layers, we devise fully connected attention (FCA), which breaks the convolutional constraint on spatial location with the assistance of full connection.
4. We propose a feature self-collaboration head (FSCHead). The low-level features that keep the spatial details are only for localization while the deep maps which refine the semantic information after multi-computation are merely for classification. FSCHead can remove redundancy in such a very concise way.

The remainder of this paper is organized as follows. Section 2 presents the related work. Section 3 introduces the details of our method. Experiments and analyses are discussed in Section 4. Finally, the paper is concluded in Section 5.

## 2. Related Work

Early methods based on manual observation and sensors have limited monitoring scope. With the evolution of smart mobile devices, machine vision-based smoke detection has been researched widely in recent years. Especially for neural network-based smoke detection, a series of novel model frameworks and advanced strategies have emerged.

### 2.1. Smoke Detection Algorithm

There are two main categories of prevalent smoke detection approaches. One is traditional descriptors based on hand-crafted features, and the other relies on deep learning [18]. Appana et al. [19] detected smoke based on its dispersion, color, and semi-transparency characteristics extracted from optical flow patterns and spatiotemporal energy. Filonenko et al. [20] took full advantage of shape and color information to evaluate the probability that a pixel belonged to the fluid smoke region. Prema et al. [21] examined distinctive texture attributes of smoke regions which were extracted by the co-occurrence of hamming distance-based local binary pattern (CoHDLBP) and co-occurrence of local binary pattern (CoLBP), including homogeneity, energy, correlation, and contrast. With the popularity of deep convolutional networks, smoke detection based on them gradually started to be explored. Hashemzadeh et al. [22] introduced a hybrid algorithm based on deep learning and spatiotemporal features of smoke. The extracted moving regions were individually submitted to a customized convolutional neural network. Tao et al. [23] presented an attention-aggregated attribute-aware network (AANet) to merge spatiotemporal and context information and decode video attributes. Gu et al. [24] proposed a deep dual-channel neural network (DCNN). The first subnetwork extracted details of smoke and the base information was captured in the second subnetwork, such as texture and contours, respectively. Almeida et al. [25] designed a lightweight convolutional neural network (CNN) for wildfire detection combined with edge computing devices. Mukhiddinov et al. [26] gave a wildfire smoke detection system using unmanned aerial vehicle (UAV) images based on an optimized YOLOv5. Saydirasulovich et al. [27] introduced the BiFormer attention mechanism in an enhanced YOLOv8 model to obtain accurate smoke detection in UAV images. Munsif et al. [28] designed an efficient deep learning model of only 9 M that can be easily deployed on resource-constrained devices. Tao et al. [29] devised a channel-enhanced spatiotemporal network (CENet) for recognizing industrial smoke emissions. A new loss function and several channel-enhanced modules ensured sufficient supervision information. Chen et al. [30] put forward an end-to-end deep neural network called DesmokeNet to remove the smoke by a two-stage recovered pipeline. Deep learning-based methods have achieved better performance than traditional techniques for smoke detection. However, due to the randomness of smoke shapes, intraclass variations, obstructions, and clutters, the pursuit of elaborate network structures while ignoring the essential attributes of smoke restricts the network’s generalization ability.

### 2.2. Cross-Layer Application Algorithm

Cross-layer modules can acquire the nonlocal associations of each layer and further strengthen the smoke-relevant feature through the aggregation and balance between the global and local contexts [31]. Li et al. [32] designed a smoke recognition model based on a detection transformer (DETR). Cross-layer deformable attention was used in the encoder-decoder structure to speed up the convergence process. Tao et al. [33] proposed a smoke density estimation network (SDENet) to explicitly analyze channel interdependencies and decode four-layer features. Its progressive cross-layer connectivity primarily relied on attention mechanisms. Zhang et al. [34] designed a multi-scale convergence coordinated pyramid network with mixed attention and fast-robust NMS (MMFNet). It combined dual attention and coordinated convergence module to refine the pyramid network and then smoke features were organized at multiple scales. Zhan et al. [35] devised an adjacent layer composite network based on a recursive feature pyramid with deconvolution and dilated convolution and global optimal non-maximum suppression (ARGNet) to monitor smoke. It improved FPN into a recursive pyramid with deconvolution and dilated convolution (RDDFPN) to achieve cross-layer multi-scale fusion. Yuan et al. [36] stacked some encoder-decoder structures and presented a wave-shaped neural network (W-Net). Cross-layer connections were employed between the peaks and troughs which contained abundant localization and semantic information, respectively. Tao et al. [37] presented a dual-branch smoke segmentation model SmokeSeger coupling a Transformer branch and a CNN branch. There are four cross-connections between the two branches to enhance the expression of global and local content. Yuan et al. [38] proposed a classification-assisted gated recurrent network (CGRNet) for smoke segmentation, where a multi-scale context contrasted local structure (MCCL) and a dense pyramid pooling module (DPPM) took similar cross-layer processing of feature maps for each layer via conventional convolution. In addition, Song et al. [39] introduced a cross-layer semantic guidance module (CSGM) to guide the shallow feature layer via deep-level information and then gave a cross-layer semantic guided network (CSGNet) based on YOLOv6. Zhang et al. [40] designed an efficient detection network based on cross-layer feature aggregation (CFANet). It integrated multi-scale features based on the avoidance of semantic gaps and compensated for the defect of layer-by-layer feature transfer that only focuses on the previous layer. It is obvious that existing cross-layer connections are used to complement global and local features and facilitate multi-scale detection, including attention mechanisms and pyramid networks.

In this paper, considering that the shallow layers contain rich spatial details and the deep layers extract precise semantic information after more computation, we propose a lightweight CLSANet by biased cross-layer connections between low-level texture features and high-level classification information.

## 3. Proposed Method

CLSANet considers the essential attributes of multi-level features for adaptive hierarchical reweighting during aggregation, and cross-layer connections are made in a biased way. In this section, the overall architecture of the CLSANet model is presented first. Then we introduce the details of three novel modules, SPM, TFM, and FSCHead, respectively. In particular, SPM is applied to the backbone, TFM is embedded at the end of feature encoding, and FSCHead directly is the head of our whole network. These novel cross-layer connection structures effectively prevent the dilution of meaningful information and pursue accurate smoke detection. Finally, we briefly describe the loss function for training.

### 3.1. Whole Network Architecture

**Figure 2:**
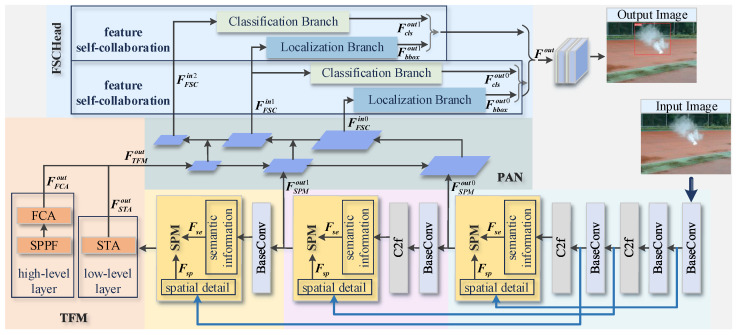
The whole network architecture of CLSANet. For catering to the different functions of high- and low-level features, cross-layer connections have divergent preferences during progressive feature fusion, allowing precise smoke detection. The backbone features are subjected to spatial restoration across four scales by SPM before being output into the pyramid PAN. The deepest coding layer also suffers from the TFM module to further complement its texture details. On the three outputs from the PAN, FSCHead carries out self-collaboration between adjacent layers for the localization and classification tasks.

We propose a lightweight CLSANet for multi-scale convergence of smoke features with different preferences, and Figure 2 shows its overall architecture. Specifically, besides the usual convolutional operation, C2F block [41], and path aggregation network (PAN) [42], it also contains three novel modules including SPM, TFM, and FSCHead. As we can see, in the backbone, the three features $F_{SPM}^{out0}$, $F_{SPM}^{out1}$, and $F_{TFM}^{out}$ are all calculated by the cross-layer fusion in the spatial perception SPM before being fed into the PAN. In SPM, the feature maps are combined with texture details mined from lower layers four times larger than themselves to enhance and integrate the selective information. Indirect feature exchange between distant layers ensures that low-level context can be dynamically preserved until the final output layer. The highest feature, $F_{TFM}^{out}$, also undergoes the texture federation TFM. This is because the deep layers experience more convolutional computation and correspondingly need to be supplemented with more underlying details. TFM aggregates the low-level spatial texture attention STA with the high-level fully connected attention FCA after spatial pyramid pooling-fast (SPPF) [43] processing to gain access to rich contexts. Subsequently, FSCHead performs a self-cooperation mechanism between neighboring layers on the three outputs of the PAN, $F_{FSC}^{in0}$, $F_{FSC}^{in1}$, and $F_{FSC}^{in2}$. The localization and classification tasks perform adjacent layer cooperation via deep decoupling.

### 3.2. Spatial Perception Module

**Figure 3:**
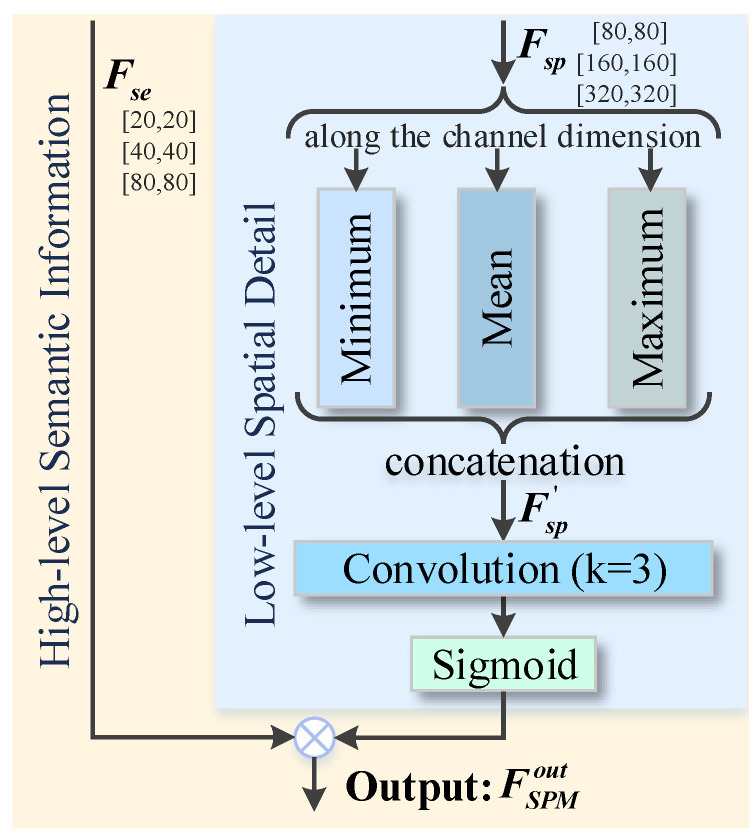
The detailed illustration of the proposed SPM. It reinforces deep feature $F_{se}$ by spatial information $F_{sp}$ from low layers across four scales, mitigating the dissipation of underlying attributes during encoding.

As the network deepens, the receptive field is gradually getting larger and the semantic expression capability is also enhanced. But it also reduces the resolution of feature maps and lots of spatial details become blurred [44]. Therefore, we design the SPM to perform cross-layer feature supplementation, as shown in Figure 3. It is employed to the input feature maps of pyramidal networks to enhance their spatial perception across four scales.

It is well known that the common smoke usually has two colors, black and white, and thus SPM simultaneously extracts both the maximum and minimum values on the shallow layer. In addition, because the pixel values of blurred boundaries and smoke regions are susceptible to the influence of ambient colors, SPM also takes the average operation to preserve the contextual details of smoke. Then after minimum, mean, and maximum operations, the three texture features are refined by a convolution with kernel 3. At last, the low-level spatial guidance maps are formed by a sigmoid function, which are loaded on the deep layers to bridge the loss of texture details. It is interesting to note that there is a fourfold difference in scale between the feature maps of the semantic and spatial layers in SPM. Such cross-layer connections allow texture information to be dynamically retained in each output layer of the network backbone, effectively addressing the issue of dissipation of contextual details during the feature encoding process.

As previously mentioned, when the low-level spatial feature is denoted as $F_{sp}$, the high-level semantic input is recorded as $F_{se}$, and the acquired intermediate feature containing textures by minimum, mean, and maximum operations is noted as $F_{sp}^{\prime }$, the output of SPM $F_{SPM}^{out}$ can be formulated as:

(1) $$\left\{\begin{array}{c} F_{sp}^{\prime }=Cat\left[min\left(F_{sp}\right),mean\left(F_{sp}\right),max\left(F_{sp}\right)\right]\\ F_{SPM}^{out}=F_{se}⊗\sigma \left(Conv\left(F_{sp}^{\prime }\right)\right) \end{array}\right.,$$

where minimum, mean, and maximum operations are along the channel dimension, Cat denotes the concatenation, Conv means the convolution with a kernel size of 3, σ is the sigmoid function, and ⊗ denotes the element-wise multiplication.

### 3.3. Texture Federation Module

**Figure 4:**
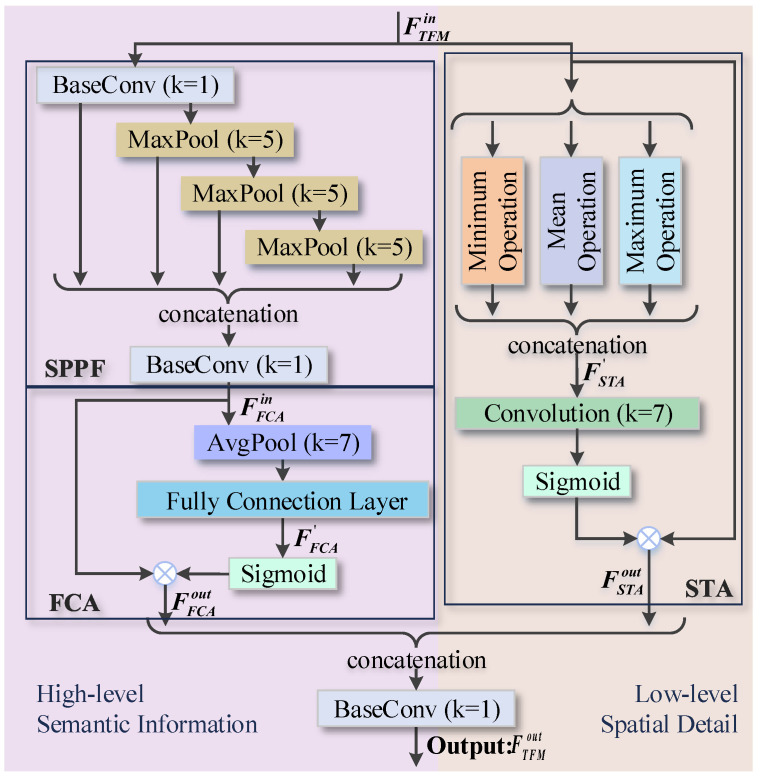
The elaborated structure of the TFM module. The STA is to preserve valuable texture details on the low-level path and the fully connected attention in FCA can reinforce the meaningful semantics of high-level layers.

Many detailed features gradually dissipate after multi-layer convolutional operations. For this reason, we design the spatial perception SPM in the backbone. But at the deepest layer of feature encoding, this issue progressively accumulates, and it is insufficient to compensate for spatial details by only relying on SPM. Therefore, we devise the texture federation TFM after SPM modification. It is arranged in the last layer of the backbone to reinforce the semantic features and further supplement the meaningful spatial details faded in the deep network maps. The elaborate structure of TFM is illustrated in Figure 4. Following the cross-layer design, STA is applied to preserve valuable low-level texture details. As for the high-level features, after the adaptive dimension of the SPPF structure, they are input into the FCA to strengthen the deep semantic information through fully connected attention. The final low- and high-level features are integrated and exported as the deepest feature encoding.

We first describe the specific process of the low-level network path. Smoke, as a salient target with fuzziness, we similarly introduce the minimum, mean, and maximum values to perceive texture. Specifically, when the input feature of the TFM is notated as $F_{TFM}^{in}$, the preliminary texture-aware feature $F_{STA}^{\prime }$ is obtained by concatenating the results of the minimum, mean, and maximum computations. Then the $F_{STA}^{\prime }$ is subjected to a convolution operation with kernel 7 and a sigmoid function to obtain the final smoke spatial filter, which will be used to filter out the invalid noise and selectively enhance the meaningful texture details in input $F_{TFM}^{in}$. The output of STA module $F_{STA}^{out}$ can thus be expressed as:

(2) $$\left\{\begin{array}{c} F_{STA}^{\prime }=Cat\left[min\left(F_{TFM}^{in}\right),mean\left(F_{TFM}^{in}\right),max\left(F_{TFM}^{in}\right)\right]\\ F_{STA}^{out}=F_{TFM}^{in}⊗\sigma \left(Conv\left(F_{STA}^{\prime }\right)\right) \end{array}\right.,$$

where minimum, mean, and maximum operations are along the channel dimension, Cat denotes the concatenation, Conv means the convolution with a kernel size of 7, σ is the sigmoid function, and ⊗ denotes the element-wise multiplication. Note that the convolutional kernel size here is 7, which is because the TFM is applied to the highest layer of the backbone, and a larger receptive field can drum up the development of global contextual dependencies.

As for the high-level branch, the characteristic flow passes through SPPF and FCA sequentially. The SPPF structure [43] enables adaptive dimension generation via multi-scale spatial containers with little increase in computational effort. When the input is denoted as $F_{TFM}^{in}$, the output $F_{SPPF}^{out}$ of SPPF can be simply obtained by:

(3) $$\begin{array}{c} {Fd}_{SPPF}^{out}=BConv(Cat[BConv\left(F_{TFM}^{in}\right),max\left(BConv\left(F_{TFM}^{in}\right)\right),\\ \;max\left(max\left(BConv\left(F_{TFM}^{in}\right)\right)\right),max\left(max\left(max\left(BConv\left(F_{TFM}^{in}\right)\right)\right)\right)]), \end{array}$$

where BConv means the base convolution including convolution, batch normalization, and SiLU activation function, Cat represents the concatenation, and max means the maximum computation with a kernel of 5 × 5.

Next, the $F_{SPPF}^{out}$ serves as the input for our FCA structure and is denoted as $F_{FCA}^{in}$. One distinct difference between FCA and existing image attention approaches is that it breaks through the spatial location constraints caused by 2D convolution and instead takes advantage of only one fully connected layer. In FCA, the $F_{FCA}^{in}$ first undergoes the average pooling to refine its spatial information, and then a simple one-layer full connection, where the operation objects of the neurons are the individual channels in the original image feature maps, reweights the global channels to introduce more possible feature representation. The obtained channel mask at this point is denoted as $F_{FCA}^{\prime }$, and immediately after it is imported into the activation function for final channel scores. The output $F_{FCA}^{out}$ of the FCA is derived by multiplying the initial input $F_{FCA}^{in}$ with the channel scores, and it is formulated as:

(4) $$\left\{\begin{array}{c} F_{FCA}^{\prime }=FC\left(avg\left(F_{FCA}^{in}\right)\right)\\ F_{FCA}^{out}=F_{FCA}^{in}⊗\sigma \left(F_{FCA}^{\prime }\right) \end{array}\right.,$$

where avg denotes the adaptive average pooling, FC means the linear fully connection, σ is the sigmoid function, and ⊗ denotes the element-wise multiplication.

Finally, to recover the spatial context details in the top-level encoding, we directly concatenate the $F_{STA}^{out}$ after spatial texture attention on the low-level pathway and the $F_{FCA}^{out}$ after fully connected attention on the high-level pathway, and then modify the channel numbers via a convolution operation so that the size of the input and output feature maps of the whole texture federation TFM module can be consistent. Therefore, the output feature $F_{TFM}^{out}$ of TFM can capture richer smoke context and the process can be defined as:

(5) $$\left\{\begin{array}{c} F_{STA}^{out}=STA\left(F_{TFM}^{in}\right)\\ F_{FCA}^{in}=SPPF\left(F_{TFM}^{in}\right)\\ F_{FCA}^{out}=FCA\left(F_{FCA}^{in}\right)\\ F_{TFM}^{out}=BConv\left(Cat\left[F_{STA}^{out},F_{FCA}^{out}\right]\right) \end{array}\right.,$$

where Cat denotes the concatenation operator and BConv means the base convolution.

### 3.4. Feature Self-Collaboration Head

**Figure 5:**
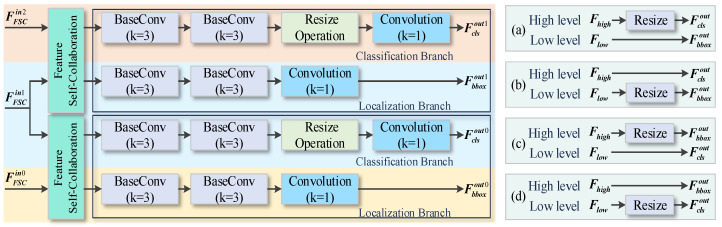
The specific design of FSCHead. Considering the decoupling of detection tasks on different branches, there are four strategies (**a**–**d**) available, and strategy (**a**) attains the best results consistent in both theory and experiment. As a result, FSCHead is designed to use high-level semantic features solely for smoke classification, while the low-level spatial details are only adopted for smoke localization. This cross-layer connection effectively removes redundant noise and refines the smoke localization and classification information.

After going through the feature pyramid, the network holds three branches at distinct scales. The conventional detection head, either anchor-based [45] or anchor-free [46], carries out the localization and classification tasks simultaneously, resulting in little communication between the different paths. Several studies [47,48,49] indicate that high-level features in deep layers encode the semantic information and acquire an abstract description of smoke, while low-level features in shallow layers retain spatial details for rebuilding the smoke boundaries. We hence propose the feature self-collaboration FSCHead, as presented in Figure 5. With cross-layer cooperation, the high-level paths are used only for the classification task, and the low-level layer is employed merely for smoke localization.

The essential idea of our FSCHead module is to adapt the feature computation to fit the appropriate detection task based on the attribute preferences of different layers, instead of indiscriminately conducting localization and classification. Here are four exclusive strategies, as displayed in Figure 5a–d. Figure 5a,b classify smoke on the deep layers which are rich in semantics, and localization is performed on the low layers which contain spatial details. Due to the scale disparity on the respective branches, Figure 5a adjusts the classification branch to match the scale of the localization branch via up-sampling, and Figure 5b tunes the scale of the localization branch to be consistent with that of the classification branch by down-sampling. In contrast, Figure 5c,d implement smoke localization on the high level and the classification task is deployed on the low level. The former produces high-resolution feature maps after up-sampling the localization branch, while the latter down-samples the classification information and thus exports low-resolution detection maps.

In addition, referring to the anchor-free mechanisms [50] and taking into account the real-time requirements for smoke detection, each branch is concretely explored. The specific classification branch and localization branch are composed of two layers of base convolution and one 2D convolution. The base convolution with a kernel of 3 is designed to reinforce the comprehension of the features and recover discriminative smoke information. The mere 2D convolution, whose kernel size is 1, adaptively modifies the channel number of the features according to the assigned task. Figure 5a achieves the best results both theoretically and practically, and such feature self-collaboration mechanism with high layers for classification and low layers for localization can simply and directly eliminate the redundancy and preserve the meaningful smoke features. Denote the three inputs of different scales of FSCHead as $F_{FSC}^{in0}$, $F_{FSC}^{in1}$, and $F_{FSC}^{in2}$, respectively. The classification and localization outputs of the low-level layers are designated as $F_{cls}^{out0}$ and $F_{bbox}^{out0}$, and $F_{cls}^{out1}$ and $F_{bbox}^{out1}$ separately represent the corresponding outputs on the high-level branches. The total output is noted as $F^{out}$, and then the transfer process of features in FSCHead can be described by:

(6) $$\left\{\begin{array}{c} F_{cls}^{out0}=Conv\left(Rs\left(BConv\left(BConv\left(F_{FSC}^{in1}\right)\right)\right)\right)\\ F_{bbox}^{out0}=Conv\left(BConv\left(BConv\left(F_{FSC}^{in0}\right)\right)\right)\\ F_{cls}^{out1}=Conv\left(Rs\left(BConv\left(BConv\left(F_{FSC}^{in2}\right)\right)\right)\right)\\ F_{bbox}^{out1}=Conv\left(BConv\left(BConv\left(F_{FSC}^{in1}\right)\right)\right)\\ F^{out}=Cat\left[F_{cls}^{out0},F_{bbox}^{out0},F_{cls}^{out1},F_{bbox}^{out1}\right]) \end{array}\right.,$$

where BConv means the base convolution, Conv means the convolution with a kernel of 1, Rs denotes the up-sampling, and Cat denotes the concatenation operator.

### 3.5. Hybrid Loss Function

In our network, a hybrid loss function is introduced to access the gap between the predicted results and the ground truths and direct the subsequent training. CLSANet has two computational outputs for each smoke target, the classification branch and the localization branch, and the total loss $L_{Loss}$ is made up of three parts. The classification loss $L_{cls}$ is computed on the classification branch, and the regression loss $L_{reg}$ and confidence loss $L_{dfl}$ are derived from the localization branch. The total loss $L_{Loss}$ is expressed as:

(7) $$L_{Loss}=\sum_{i=1}^{n}L_{loss}^{i}=\sum_{i=1}^{n}\left(L_{cls}^{i}+\alpha L_{reg}^{i}+\beta L_{dfl}^{i}\right),$$

where α and β are the weight parameters, i∈[0,1] denotes the two different forward paths in the head, and $L_{loss}^{i}$ signifies the sum of losses on the forward path *i*.

Specifically, classification in smoke detection is a binary task, and a binary cross-entropy loss (BCE) is adopted to guide its optimization. BCE is easy to deploy and has a high computational efficiency. As for smoke localization, it is essentially a regression task, and a complete intersection over union loss (CIOU) is employed to penalize the inconsistent results on it. CIOU takes into account the intersection over union, centroid distance, and relative proportions between the predicted and true values. It is suitable for smoke detection tasks that have various shapes. Furthermore, since smoke detection is binarised and its distribution is highly steep, a distributional focal loss (DFL) is thus implemented in the regression branch to further refine the coordinates of the detection boxes after decoding their integrals. As a result, we can acquire the $L_{cls}^{i}$, $L_{reg}^{i}$, and $L_{dfl}^{i}$ losses by:

(8) $$\left\{\begin{array}{c} L_{cls}^{i}=BCE\left(cls^{i},cls_{gt}^{i}\right)\\ L_{reg}^{i}=CIOU\left(bbox^{i},bbox_{gt}^{i}\right)\\ L_{dfl}^{i}=DFL\left(bbox^{i},bbox_{gt}^{i}\right) \end{array}\right.,$$

where i∈[0,1] means the two different forward paths in the head, cls and bbox denote the predicted classification probability and bbox coordinate, respectively, and $cls_{gt}$ and $bbox_{gt}$ represent the corresponding ground truths, respectively.

## 4. Experimental Evaluation

In this section, we first present the two databases used for the experiments, and immediately after, the specific training details are described. Then, we compare CLSANet with state-of-the-art object detection methods and several available smoke detection algorithms. Thirdly, an ablation analysis for each component is organized. In addition, we present the model size and time complexity to validate that the CLSANet is lightweight and efficient. Furthermore, to verify that our algorithm can accurately focus on the smoke regions, we carry out a feature visualization discussion. Finally, the detection results of our CLSANet in real scenarios are given, and we analyze the success and failure cases, respectively.

### 4.1. Database

There are two available databases used in this paper, which are the annotated real smoke database of Xi’an Jiaotong University (XJTU-RS) [51] and the real smoke and forest background database of University of Science and Technology of China (USTC-RF) [52], respectively. Their statistics are shown in Table 1.

**Figure 6:**
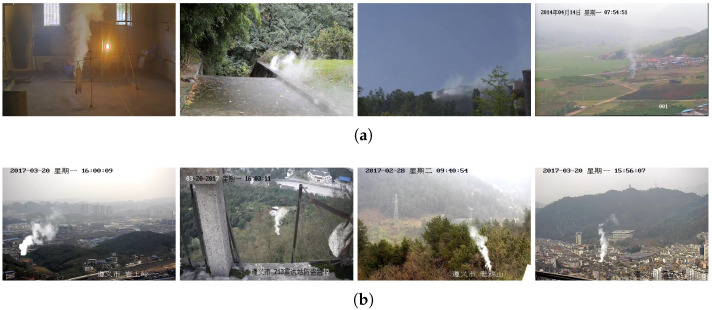
Sample images of (**a**) the XJTU-RS [51] and (**b**) the USTC-RF [52] databases. The XJTU-RS database is manually labeled from realistic images, while the USTC-RF database is synthesized from forest images and simulated smoke frames jointly.

XJTU-RS contains a total of 6845 images, all of which are taken in real scenes and annotated manually. There are 4791 images in the training set, 1369 images in the validation set, and 685 images in the test set. To make the trained model meet the requirements for practical application, XJTU-RS acquires the original images from two real but unlabeled benchmark databases: Keimyung University (KMU) [53] and University of Science and Technology of China (USTC) [52]. Their smoke images are taken in various natural scenarios, e.g., indoor smoke, forest smoke, playground smoke, and farmland smoke. To enhance the robustness of XJTU-RS, stratified random sampling is arranged in almost every scene. Finally, the smoke regions are demarcated with rectangular boxes and corresponding labels supported by a published annotation tool. Some sample images of XJTU-RS are displayed in Figure 6a, and they are manually annotated and quite realistic.

USTC-RF comprises 12,620 synthesized smoke images, out of which 3155 are selected for training, 3155 for validation, and the remaining 6310 for testing. In this dataset, in order to precisely extract the smoke, the real smoke image is photographed against a green background, and based on it, the pure smoke regions are derived. There are 2800 smoke frames selected from 10 smoke videos with green backgrounds. To further increase the smoke diversity, rendering software is employed to generate the smoke plumes prepared for the synthesized images, where the initial flow rate, airflow, illumination, and viewing angle are all set randomly. A total of 1000 synthetic smoke plumes are obtained from such simulations. At last, the smoke plumes are freely changed in shape and inserted into 12,620 forest background images at random locations. Each synthetic image includes only one wisp of smoke and the location of the smoke can be provided automatically during the insertion process. Examples of images from USTC-RF are presented in Figure 6b.

### 4.2. Implementation Details

The experiments are implemented on a PC with an Intel CPU i7-8700K @ 3.2 GHz, 16 GB of RAM, and an NVIDIA RTX2080Ti with 11 GB. Our proposed CLSANet is trained using the Pytorch framework, and it is lightweight with only 2.38 M, allowing real-time detection on such a hardware platform. In addition to the regular convolution and C2F operations, the spatial perception SPM and texture federation TFM modules are introduced in the backbone for biased cross-layer feature communication. PAN pyramid is closely followed for multi-scale feature fusion, and finally, the FSCHead deploys the feature self-cooperation mechanism on the high-level and low-level branching channels. It also applies the anchor-free mechanism to alleviate the inherent conflict between classification and localization. During the training period, CLSANet is optimized by the stochastic gradient descent (SGD) with a weight decay of 0.0005, a momentum of 0.937, and an initial learning rate of 0.01. Mosaic strategy [54] which splices four random images is exploited to augment the data and complicate the detection background. As for the sample assignment strategy, CLSANet employs the task-aligned assigner which selects positive data based on the weighted scores of classification and regression. The threshold of intersection over union for non-maximum suppression is set to 0.7 and the coefficients of classification loss, regression loss, and distributional focal loss are 0.5, 7.5, and 1.5, respectively.

To avoid overfitting, CLSANet is trained on the training database and validated on the validation database. The images for training and validation are different. Moreover, the training is automatically terminated when the network has no improvement in the last 50 epochs in training.

In this paper, we adopt widely used metrics to evaluate the performance of CLSANet, including the average precision (AP) and the average recall (AR). In addition, the average precision of detecting small ($AP_{S}$), medium ($AP_{M}$), and large ($AP_{L}$) areas are also explored to further delve into the superiority of our model. Note that the smoke regions in the USTC-RF database are all larger than the scale of the defined large area, i.e., 96 × 96. It means that the $AP_{S}$ and $AP_{M}$ are null and the values of $AP_{L}$ and AP are the same. So in the experimental results, we no longer give the $AP_{S}$, $AP_{M}$, and $AP_{L}$ on USTC-RF database.

### 4.3. Comparison with Regular Objects Detection Methods

In this section, some popular and state-of-the-art object detection methods, which are Faster R-CNN [55], SSD [12], RetinaNet-50 [13], RetinaNet-101 [13], EfficientDet [14], YOLOv5 [15], YOLOv7 [56], YOLOX [16], EdgeYOLO-Tiny [17], and EdgeYOLO-S [17], are compared with the proposed CLSANet on the same test environment. We also conducted experiments on two transformer-based networks, an improved YOLOv5 based on transformer prediction head (TPH-YOLOv5) [57] and a real-time detection transformer (RT-DETR) [58], respectively. The $AP_{S}$, $AP_{M}$, $AP_{L}$, AP, and AR of different networks on XJTU-RS database are presented in Table 2, but since the $AP_{S}$ and $AP_{M}$ are null and the values of $AP_{L}$ and AP are the same on the USTC-RF database, there are only the corresponding AP and AR for the different algorithms given in Table 3. The best results in Table 2 and Table 3 are shown in bold font.

From the experimental statistics, it is quite obvious that our algorithm achieves the best performance on both the XJTU-RS and USTC-RF databases. Specifically, benefiting from cross-layer connections, our CLSANet attains the best results on all metrics except the $AP_{S}$ on the XJTU-RS which is only 2.2% less than the optimal result of RetinaNet-50 in Table 2. The feature extraction of Faster R-CNN and SSD is just a simple stack of several convolutional layers without feature modification and supplementation among distinct levels. As a result, the AP of Faster R-CNN and SSD is 65.5% and 65.9% on XJTU-RS and 78.4% and 81.0% on USTC-RF, respectively. RetinaNet-50 and RetinaNet-101 have much better comprehensive capabilities than them. It is attributed to the residual unit in RetinaNet, which also belongs to a kind of simple cross-layer connection. What’s more, RetinaNet-101 has more residual units than RetinaNet-50. It results in a more sufficient exchange of indirect information between the distant layers, and thus RetinaNet-101 is slightly more effective than RetinaNet-50. In contrast, EfficientDet produces the lowest AP on both databases, 65.4% and 60.1%, and its AR is the second worst on XJTU-RS and the worst on USTC-RF, 69.6% and 63.7%, respectively. The bi-directional feature pyramid network (BiFPN) [59] in EfficientDet allows cross-layer fusion on feature encoding maps of the backbone output at three scales. Evidently, EfficientDet trades information only on depth-coded feature maps and it is not sufficient for the detection of fickle smoke.

As for the YOLO series, which has gained enormous popularity in recent years, it can be found that their entire performance is relatively superior. To be specific, the bottleneck cross-stage partial network (CSPNet) of YOLOv5 [60] enables cross-layer fusion of internal information through feature concatenation. Referring to VoVNet’s [61] strategy, YOLOv7 modifies the CSPNet module as an efficient layer aggregation network (ELAN) [56]. It adopts a stack structure to deepen the feature transmission path. Consequently, YOLOv7 which has more cross-layer communication convincingly behaves better than YOLOv5. Furthermore, YOLOX applies the anchor-free mechanism in its head, and such decoupling between the classification and localization is also employed in our method. As shown in Table 2, the AP of YOLOX on the XJTU-RS is relatively acceptable with 71.3%. In addition, EdgeYOLO is designed specifically for edge devices. From Table 3, the EdgeYOLO-Tiny has the sub-optimal results on the USTC-RF in terms of AP with 91.2%. It is because the EdgeYOLO is designed to further enrich the feature diversity of input images through a flexible data augmentation. The detection performance of both EdgeYOLO-Tiny and EdgeYOLO-S on the XJTU-RS and USTC-RF databases is favorable and indistinguishable.

In recent years, many teams have developed various object detection models using the transformer architecture. We compare CLSANet with two transformer-based models TPH-YOLOv5 and RT-DETR. The results are shown in Table 2 and Table 3. Since the transformer’s self-attention mechanism allows the model to simultaneously consider all positions of the input sequence and learn the dependencies between different positions, the results achieved by TPH-YOLOv5 and RT-DETR on both databases are significant. In particular, on XJTU-RS database, RT-DETR achieves the AP only second to our algorithm, which is 71.4%. The global attention in transformer allows the model to capture global dependencies, which increases the complexity while delivering fine detection performance. The number of parameters for TPH-YOLOv5 and RT-DETR are 41.49 M and 31.99 M, respectively. Compared to our CLSANet which is only 2.38 M, it is clear that transformer-based algorithms are not suitable for resource-limited edge devices.

The comparison among these object detection algorithms indicates that the smoke detection performance can be improved to varying degrees by reinforcing cross-layer feature exchange. Based on the results in Table 2 and Table 3, we can conclude that smoke detection, as a simple binary task, is not boosted as the model deepens, but relies more on the specific network design. Our CLSANet specifically devises three strategies, SPM, TFM, and FSCHead, to realize dynamic cross-layer communication with different biases based on smoke attributes. As a result, it obtains the best smoke detection results on both XJTU-RS and USTC-RF databases.

### 4.4. Comparison with Special Smoke Detection Models

To further validate the performance of the CLSANet, we select several smoke detection networks in recent years and conduct experiments both on XJTU-RS and USTC-RF. As we can see, they are DCNN [24], W-Net [36], SASC-YOLOX [51], Deep CNN [62], STCNet [63], and MVMNet [64], respectively. Since most available warning networks are only intended to classify smoke, for the sake of a fair comparison, we uniformly substitute a conventional anchor-free head for the fully connected layer at the end of the smoke classification network. The corresponding results on XJTU-RS and USTC-RF are listed in Table 4 and Table 5, respectively, with the best results in bold font.

Specifically, Cao et al. [63] devised a novel spatiotemporal cross network (STCNet). It involves a spatial pathway to capture the smoke texture and a temporal route to extract motion information. It can be seen that STCNet has the most poor detection performance among these methods on the two databases, with AP of 57.2% and 70.9%, and AR of 63.5% and 75.5%, respectively. This may be because the STCNet, as a residual frame structure for video smoke detection, is not appropriate for training sets which are labeled image by image in XJTU-RS and USTC-RF.

An energy-efficient deep CNN was proposed by Khan et al. [62]. It slightly outperforms STCNet in experiments and is the second worst on XJTU-RS. However, Deep CNN has only simple convolution and fully connected layers but without any cross-layer communication, which results in the loss of much critical smoke information as the network deepens, especially small smoke features. Consequently, Deep CNN is the least in terms of $AP_{S}$ with only 36.8%.

In order to enhance the cross-layer feature integration, Yuan et al. [36] designed a wave-shaped neural network (W-Net). It copies and resizes the encoding outputs, and then concatenates them between peaks and troughs. As shown in Table 4 and Table 5, the smoke detection performance of the W-Net is further improved. However, its cross-layer communication is simply splicing and does not individually utilize the advantages of different layers.

Gu et al. [24] gave a deep dual-channel neural network (DCNN). The texture details and base information of the smoke are derived from the two channels separately, which can alleviate the dissipation of smoke details. It is more capable than W-Net and has AP of 66.0% and 85.3% on XJTU-RS and USTC-RF, respectively.

In addition, a multi-orientated detection based on a value conversion-attention mechanism module and mixed-NMS (MVMNet) was proposed by Hu et al. [64]. As shown in Table 4 and Table 5, the AP of MVMNet is only 1.9% and 5.6% less than that of our CLSANet on the two databases, respectively. It is because MVMNet deploys a conversion-attention mechanism to reinforce the spatial and textural information of smoke.

Wang et al. [51] presented a self-attention and self-cooperation YOLOX (SASC-YOLOX). It obtains attention weights from the low layers and realizes feature reinforcement between the high-level semantics and the low-level spatial information. Such cross-layer connection is endowed with preliminary feature complementary, and accordingly, it is second only to the proposed CLSANet in terms of comprehensive capability in experiments.

Lastly, CLSANet attains the best detection results on both XJTU-RS and USTC-RF, with AP of 73.3% and 94.4%, and AR of 72.1% and 95.3%, respectively. It justifiably validates the effectiveness of our optimization strategies. Cross-layer communication can enhance the network’s ability to extract smoke features, and this individualized transfer between high and low layers with bias can further reduce noise and refine valuable smoke features.

### 4.5. Ablation Study

We first analyze the effectiveness of each module, that is, SPM, TFM, and FSCHead. The ablation experiments are shown in Table 6. Then, a clear comparison between the convolutional block attention module (CBAM) and our proposed SPM and TFM modules is provided in Table 7. Finally, four strategies of feature self-collaboration mechanism are compared to demonstrate the FSCHead design and the corresponding results are provided in Table 8. All ablation experiments are conducted on both XJTU-RS and USTC-RF databases, and the best results are denoted in bold font.

#### 4.5.1. Effectiveness of SPM

As shown in Table 6, the addition of SPM obviously brings a boost in terms of all indicator scores. When the model is only equipped with SPM, the AP increases by 5.29% and 2.77% from baseline on both databases and the AR is improved by 4.28% and 2.14%, respectively. When using SPM as a variable, by comparing (1) and (2), (3) and (5), (4) and (6), and (7) and (8), the AP scores are improved with a margin of 5.29%, 1.54%, 1.82%, and 1.52% on XJTU-RS, respectively, and 2.77%, 1.42%, 2.30%, and 1.51% on USTC-RF, respectively. In particular, there is an increase of 17.54% in $AP_{S}$ on XJTU-RS database, which demonstrates that the small-scale smoke features are effectively preserved. The SPM can retain the low-level spatial details through three cross-layer connections until the highly encoded semantic information.

#### 4.5.2. Effectiveness of TFM

In the baseline of the experiment (1), the highest layer of the backbone is a spatial pyramid pooling (SPP) module. Through comparing (1) and (3), a gain of 4.9% and 1.89% in AP is obtained, respectively, with 4.13% and 1.42% of the improvement occurring in AR. Through comparing (1) and (3), the gains of AP are obtained as 4.9% and 1.89%, and 4.13% and 1.42% improvement occurs in AR, respectively. When comparing (2) and (5), (4) and (7), and (6) and (8), the network applied with the TFM structure convincingly has better metrics on XJTU-RS and USTC-RF. It verifies that TFM exploits FCA and STA attention to perform cross-layer feature filtering along two different dimensions at the deep encoding layer and then achieve the perception of meaningful information.

#### 4.5.3. Effectiveness of FSCHead

Experiment (4) is optimized with FSCHead only. When compared to (1), it increases $AP_{S}$, $AP_{M}$, $AP_{L}$, AP, and AR by 13.90%, 6.58%, 4.76%, 5.14%, and 4.72% on XJTU-RS, respectively, and AP and AR are improved by 1.55% and 1.31% on USTC-RF, respectively. Taking FSCHead as a variable, by comparing (1) and (4), (2) and (6), (3) and (7), and (5) and (8), AP obtains an absolute gain of 1.55%, 1.08%, 1.31%, and 1.40% on the synthetic USTC-RF, respectively. And it mitigates the dissipation of small-scale smoke details during transmission. As shown in Table 6, $AP_{S}$ intuitively increases by 13.90%, 8.41%, and 12.17% on XJTU-RS by the comparison between (1) and (4), (3) and (7), and (5) and (8), respectively. It sufficiently proves that the feature self-collaboration mechanism of FSCHead can preserve the important features and remove redundancy through cross-layer communication.

#### 4.5.4. Comparison with CBAM

CBAM is a simple but effective attention module for feed-forward convolutional neural networks [65]. It optimizes the network in both channel and spatial dimensions, allowing the improved model to acquire meaningful features from both channel and spatial perspectives. Like cross-layer connection, it is a common way to enhance feature extraction. To further validate the effectiveness of our cross-layer connection strategy in smoke feature extraction and detection, we replace the SPM and TFM modules in CLSANet with CBAM in turn for experiments, and the comparison results are listed in Table 7. The first CBAM-equipped model achieves favorable results on both XJTU-RS and USTC-RF databases and even outperforms some conventional object detection networks, such as RetinaNet-101 [13] and YOLOv5 [15]. However, the overall performance of our proposed CLSANet is still superior to that of CBAM-equipped networks. Except for the AP*_S_* on XJTU-RS, which is second only to the optimal result of 53.7%, CLSANet outperforms the CBAM-equipped model in all metrics.

#### 4.5.5. Configurations of FSCHead

To determine the appropriate FSCHead design for retaining meaningful smoke features, the experiments are carried out sequentially on four different feature self-collaboration strategies, which are FSCHead (a) to (d), as illustrated in Figure 5a–d. The test results are reported in Table 8, and it can be easily noticed that the detection performance of FSCHead (c) and (d) significantly lags behind FSCHead (a) and (b) on both XJTU-RS and USTC-RF. It may be because the higher layers can accurately extract semantic information after more computation, which is beneficial for classification, whereas the lower layers which have smaller receptive fields can retain spatial details and thus guide the localization. The performance of FSCHead (a) is again superior to that of FSCHead (b), and the best AP on the two databases are 71.6% and 91.5%, respectively. It also suggests that the more information contained in the feature map, the stronger the network is. The variants of the four different configurations confirm that the high-level branch for classification and the low-level branch for localization is an adaptive scheme to the smoke detection task and that the design of our FSCHead plays an important role in CLSANet.

### 4.6. Model Size

The smoke warning is usually used in surveillance systems, so the number of parameters and computational complexity are crucial metrics to evaluate the suitability of the model for resource-constrained edge devices. In this section, we compare CLSANet with some conventional methods in terms of AP, AR, the number of parameters (Params), and frames per second (FPS). The comparative results on XJTU-RS and USTC-RF are shown in Table 9, with the best values in bold.

It is obvious that the two-stage Faster R-CNN [55] has the most parameters. Accordingly, its computational speed is the slowest among all algorithms, with only 7.790 FPS and 3.849 FPS on XJTU-RS and USTC-RF, respectively. The bad capability does not allow Faster R-CNN to be applied to smoke detection in the real world. SSD [12] and RetinaNet-50 [13] mainly rely on stacking layers to extract features. This simple deepening of the network increases the number of parameters while increasing the computational complexity. Therefore, SSD and RetinaNet-50 are unsuitable for polymorphic smoke. In particular, the backbone of RetinaNet-50 adopts the residual network (ResNet), which is composed of multiple densely connected convolutional layers. With 36.33 M parameters, its FPS on the two databases are only 8.421 and 6.917.

In YOLO series, such as YOLOv7 [56], YOLOX [16], and EdgeYOLO [17], the network design becomes more exquisite, and cross-layer connections are gradually employed to enhance feature exchange over long distances. Therefore, their accuracy and model size are further developed. The EdgeYOLO-Tiny is only 5.81 M and its AP and AR on USTC-RF are only second best to our algorithm. The proposed CLSANet achieves consistently optimal results among all the methods, with the best accuracy and recall on both XJTU-RS and USTC-RF. What’s more, its model size is only 40.96% of that of the sub-optimal EdgeYOLO-Tiny, i.e., 2.38 M. It verifies the cross-layer connection can ensure the network has superior smoke awareness even with a few parameters. As for the detection speed, it has the optimal FPS on XJTU-RS with 239.981 and the sub-optimal 219.491 on the USTC-RF database, second only to YOLOX. Our algorithm can fully satisfy the real-time requirement of smoke alarm systems. We can declare that the CLSANet is the best candidate to detect smoke in constrained environments and resource-limited equipment.

### 4.7. Feature Visualization

**Figure 7:**
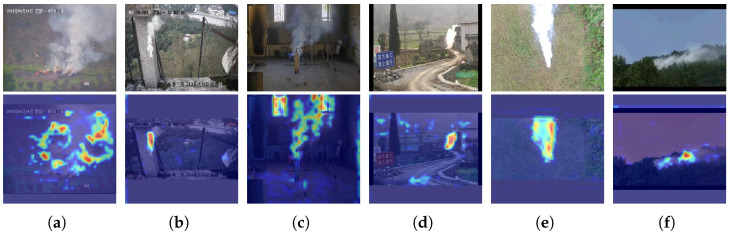
Feature visualization of the proposed CLSANet. (**a**–**f**) The first line is the original input and the second line is the corresponding visualization map. The CLSANet can accurately perceive smoke in different scales and forms.

To validate the accuracy of CLSANet’s attention to specific smoke areas, we performed a visualization analysis. The input samples and corresponding results are presented in Figure 7. Because the images in the XJTU-RS database are realistic and cover complex scenes, the weights used for the visualization are obtained on XJTU-RS.

We adopt the classical gradient-weighted class activation mapping (Grad-CAM) method for visualization [66]. It uses the score values corresponding to the target categories to compute the gradient of the feature map, thus generating a heat map to show the important image regions in network prediction. As we can see, the proposed CLSANet model can accurately perceive smoke at various scales, such as large smoke in Figure 7a and small smoke in Figure 7d. Moreover, different concentrations of smoke can also be well focused by CLSANet, such as light smoke in Figure 7c and thick smoke in Figure 7e.

### 4.8. Practical Application

**Figure 8:**
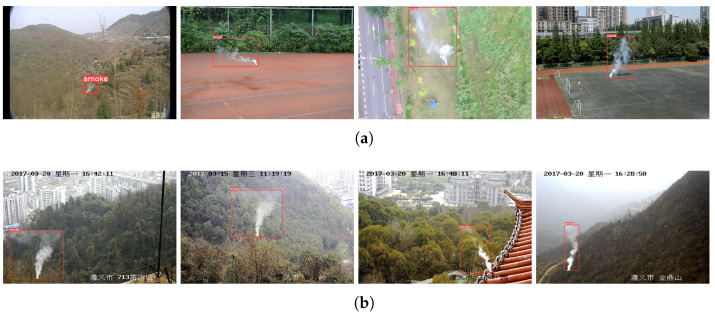
Visual detection for smoke images on (**a**) XJTU-RS and (**b**) USTC-RF databases. It can be seen that CLSANet has excellent detection performance on the test images.

**Figure 9:**
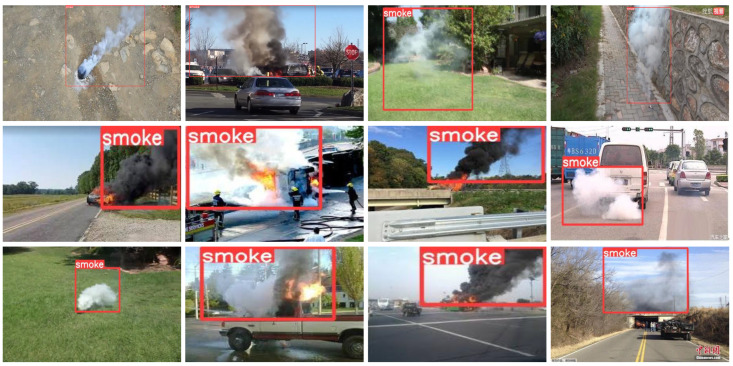
Visual detection for smoke images in the real application. These images are randomly downloaded from the Internet and untrained, and our CLSANet can still accurately monitor smoke.

Smoke detection is a key part of disaster and incident monitoring, so to meet the requirements of real-world applications, CLSANet needs to have favorable reliability in both the trained databases and the untrained scenarios. To validate that the training of the model is not overfitting and that the CLSANet we obtained has a strong generalization ability, we first randomly select some images from both XJTU-RS and USTC-RF and present their detection results in Figure 8. Then, we download several smoke images from the Internet and the results of CLSANet are visualized in Figure 9.

There are four representative smoke images selected from XJTU-RS here and their visualization results of the CLSANet are shown in Figure 8a. As we can see, CLSANet is not affected by the surrounding gray blurred mountains and exhibits remarkable detection of small-scale smoke in the first image. The second and third images revealed that CLSANet can accurately perceive the smoke although it is faint and blended with the background. In addition, as illustrated in the last two, there is no doubt that CLSANet has robust discrimination of obvious white smoke and large regions of smoke blocks. As for USTC-RF, as presented in Figure 8b, we chose the synthetic images from diverse scenarios. It is clear that our CLSANet is able to precisely localize the smoke plume under different drift directions and diffusion conditions.

To further validate the generalization ability of our network, in Figure 9, we download smoke with different morphologies from the Internet, where the images in the first row have high resolution. It can be seen that CLSANet has satisfactory smoke detection performance in these untrained natural environments, including smoke in black and white, smoke in thick and light, smoke at varied scales, smoke with distinct diffusion directions and morphologies, and images of different capture quality. It is mainly because the cross-layer connections in CLSANet retain as much meaningful smoke information as possible and cut down the interference of noise under variable conditions. All in all, based on these intuitive results, we can claim that our method’s training is appropriate. CLSANet has strong robustness and remarkable performance in various smoke environments.

### 4.9. Failures Analysis

**Figure 10:**
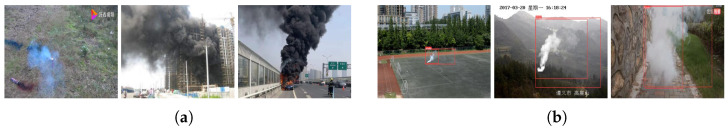
Samples of failed detection by CLSANet. These are the defective results on (**a**) blue and black smoke and (**b**) accurate smoke areas respectively.

When detecting smoke images, CLSANet inevitably makes some mistakes. We analyze the failure cases from the Internet and the training database, and some samples are displayed in Figure 10.

As we can see, there are two main categories of failure detection. In Figure 10a, CLSANet is unable to correctly identify some smoke with special colors such as blue and black, which is mainly due to the lack of corresponding smoke samples in the training database. It can be addressed by further expanding the database. In Figure 10b, the shortcoming is mainly the inaccurate delineation of the smoke region. CLSANet can successfully detect smoke in these three images, but there are overlaps between the output detection boxes. It may be due to an improper threshold of the non-maximum suppression, resulting in some overlapping frames not being completely suppressed. This can be mitigated by tuning some hyper-parameters according to the specific task. In addition, it inspires us to consider further design and refinement on the decision-making strategy of CLSANet. When the model’s calculation of the location and size of the smoke is sufficiently deterministic, it is possible to avoid generating multiple close but slightly different detection boxes.

## 5. Conclusions

Automatic smoke detection plays an essential role in monitoring fires and safeguarding the ecological environment. In this paper, we propose a lightweight CLSANet with only 2.38 M to detect smoke in real-time. The major difference with the existing algorithms is that CLSANet exploits multiple cross-layer connections with a bias towards high and low-level features. It is based on the fact that the lower layers contain richer spatial details while the higher layers have more precise semantic information. Specifically, we first design the spatial perception SPM in the backbone, which spans four scales and passes shallow features to high layers to compensate for the dissipation of spatial details. Then, the texture federation TFM is proposed and applied to repair the final feature encoding. The STA and FCA in the TFM module assist the cross-layer feature integration along the spatial and channel dimensions, where the full connection layer in the FCA breaks the constraints of spatial location imposed by convolution and facilitates the TFM to acquire more robust semantics. Finally, we propose FSCHead guided by the feature self-collaboration mechanism to preserve the significant information and eliminate redundancy in a concise way. It decouples the detection task and explicitly deploys the task of localization on low-layer branches while smoke classification on high-layer pathways. We conduct extensive experiments on the XJTU-RS and USTC-RF databases, and the results show that our CLSANet achieves state-of-the-art performance compared to both regular object detection algorithms and specialized smoke detection methods. Compared to the baseline, our algorithm improves the precision by 7.64% and 4.77% on XJTU-RS and USTC-RF, respectively.

In future work, our main research direction is firstly to address the existing deficiencies of CLSANet. Improve its detection ability for various colors of smoke, and optimize the size and scale of its anchor boxes to better match the smoke outside the training database. Secondly, it will be deployed to specific edge devices such as unmanned aerial vehicles (UAVs), Internet of Things (IoT) systems, and forest monitoring for field testing. CLSANet is expected to be easily transplanted to portable hardware and maturely applied to fire warnings.

## Figures and Tables

**Table 1:** Statistical data of the smoke databases.

Database	Total	Training	Validation	Testing
XJTU-RS [51]	6845	4791	1369	685
USTC-RF [52]	12,620	3155	3155	6310

**Table 2:** Experimental results of the proposed and ten state-of-the-art methods on XJTU-RS.

Method	AP*_S_*/%	AP*_M_*/%	AP*_L_*/%	AP/%	AR/%
Faster R-CNN [55]	38.5	60.2	68.0	65.5	71.2
SSD [12]	49.6	61.2	67.9	65.9	68.7
RetinaNet-50 [13]	**52.9**	63.2	71.8	68.9	70.0
RetinaNet-101 [13]	48.6	63.2	72.7	69.1	70.1
EfficientDet [14]	46.2	63.3	67.6	65.4	69.6
YOLOv5 [15]	51.3	63.1	69.0	66.1	70.3
YOLOv7 [56]	43.8	63.2	74.1	70.9	70.0
YOLOX [16]	48.1	63.2	74.6	71.3	70.1
EdgeYOLO-Tiny [17]	45.5	63.0	74.3	71.1	70.2
EdgeYOLO-S [17]	45.2	63.5	74.2	71.1	70.5
TPH-YOLOv5 [57]	51.8	63.7	74.4	71.3	71.9
RT-DETR [58]	47.0	65.1	74.3	71.4	71.0
CLSANet (ours)	50.7	**66.1**	**76.2**	**73.3**	**72.1**

**Table 3:** Experimental results of the proposed and ten state-of-the-art methods on USTC-RF.

Method	AP/%	AR/%
Faster R-CNN [55]	78.4	82.5
SSD [12]	81.0	85.2
RetinaNet-50 [13]	82.9	86.0
RetinaNet-101 [13]	86.8	90.7
EfficientDet [14]	60.1	63.7
YOLOv5 [15]	88.5	90.7
YOLOv7 [56]	90.5	92.4
YOLOX [16]	90.7	92.3
EdgeYOLO-Tiny [17]	91.2	92.8
EdgeYOLO-S [17]	91.1	92.8
TPH-YOLOv5 [57]	91.0	92.9
RT-DETR [58]	90.7	93.1
CLSANet (ours)	**94.4**	**95.3**

**Table 4:** Comparison results of different smoke detection algorithms on XJTU-RS.

Method	AP*_S_*/%	AP*_M_*/%	AP*_L_*/%	AP/%	AR/%
DCNN [24]	46.0	62.5	67.7	66.0	70.6
W-Net [36]	44.1	59.8	67.1	65.1	70.4
SASC-YOLOX [51]	**53.5**	64.8	75.5	72.7	71.4
Deep CNN [62]	36.8	56.2	65.1	62.7	67.8
STCNet [63]	37.0	49.8	60.3	57.2	63.5
MVMNet [64]	**53.5**	65.2	73.9	71.4	70.7
CLSANet (ours)	50.7	**66.1**	**76.2**	**73.3**	**72.1**

**Table 5:** Comparison results of different smoke detection algorithms on USTC-RF.

Method	AP/%	AR/%
DCNN [24]	85.3	87.6
W-Net [36]	77.0	80.7
SASC-YOLOX [51]	92.3	94.0
Deep CNN [62]	87.6	89.6
STCNet [63]	70.9	75.5
MVMNet [64]	88.8	90.7
CLSANet (ours)	**94.4**	**95.3**

**Table 6:** Ablation analysis of each component in CLSANet on XJTU-RS and USTC-RF.

No.	[SPM]	[TFM]	[FSCHead]	XJTU-RS [51]					USTC-RF [52]	
				AP*_S_*/%	AP*_M_*/%	AP*_L_*/%	AP/%	AR/%	AP/%	AR/%
(1)				43.9	59.3	71.5	68.1	67.8	90.1	91.8
(2)	✓			**51.6**	62.9	75.0	71.7	70.7	92.6	93.8
(3)		✓		45.2	63.9	74.5	71.5	70.6	91.8	93.1
(4)			✓	50.0	63.2	74.9	71.6	71.0	91.5	93.0
(5)	✓	✓		45.2	65.5	75.6	72.6	71.5	93.1	94.1
(6)	✓		✓	46.1	65.1	75.9	72.9	72.0	93.6	94.5
(7)		✓	✓	49.0	64.4	75.1	72.2	70.8	93.0	94.2
(8)	✓	✓	✓	50.7	**66.1**	**76.2**	**73.3**	**72.1**	**94.4**	**95.3**

**Table 7:** Comparison of CBAM with our proposed SPM and TFM modules on XJTU-RS and USTC-RF.

[Module 1]	[Module 2]	XJTU-RS [51]					USTC-RF [52]	
		AP*_S_*/%	AP*_M_*/%	AP*_L_*/%	AP/%	AR/%	AP/%	AR/%
CBAM [65]	CBAM [65]	**53.7**	65.1	74.7	72.0	71.6	93.3	94.6
CBAM [65]	TFM	46.4	64.9	75.7	72.7	71.7	93.7	94.7
SPM	CBAM [65]	48.1	64.6	73.9	71.3	70.9	93.4	94.7
SPM	TFM	50.7	**66.1**	**76.2**	**73.3**	**72.1**	**94.4**	**95.3**

**Table 8:** Performance of four feature self-collaboration strategies for FSCHead on XJTU-RS and USTC-RF.

Method	XJTU-RS [51]					USTC-RF [52]	
	AP*_S_*/%	AP*_M_*/%	AP*_L_*/%	AP/%	AR/%	AP/%	AR/%
FSCHead (a)	**50.0**	**63.2**	**74.9**	**71.6**	**71.0**	**91.5**	**93.0**
FSCHead (b)	45.1	63.0	**74.9**	71.4	70.3	89.1	90.4
FSCHead (c)	42.9	59.6	72.1	68.6	68.2	87.9	89.8
FSCHead (d)	45.4	60.7	72.1	68.8	68.3	88.1	89.8

**Table 9:** Study of model size and FPS for different detection networks on XJTU-RS and USTC-RF.

Method	XJTU-RS [51]			USTC-RF [52]			Params
	AP/%	AR/%	FPS	AP/%	AR/%	FPS	
Faster R-CNN [55]	65.5	71.2	7.790	78.4	82.5	3.849	41.35 M
SSD [12]	65.9	68.7	39.214	81.0	85.2	24.138	23.75 M
RetinaNet-50 [13]	68.9	70.0	8.421	82.9	86.0	6.917	36.33 M
YOLOv7 [56]	70.9	70.0	25.242	90.5	92.4	28.736	9.14 M
YOLOX [16]	71.3	70.1	232.019	90.7	92.3	**265.252**	8.94 M
EdgeYOLO-Tiny [17]	71.1	70.2	123.457	91.2	92.8	128.370	5.81 M
EdgeYOLO-S [17]	71.1	70.5	92.851	91.1	92.8	93.545	9.86 M
CLSANet (ours)	**73.3**	**72.1**	**239.981**	**94.4**	**95.3**	219.491	**2.38 M**

## Data Availability

The data are available upon reasonable request from the corresponding author.
